# Fine Particulate Matter and Incident Cognitive Impairment in the REasons for Geographic and Racial Differences in Stroke (REGARDS) Cohort

**DOI:** 10.1371/journal.pone.0075001

**Published:** 2013-09-25

**Authors:** Matthew Shane Loop, Shia T. Kent, Mohammad Z. Al-Hamdan, William L. Crosson, Sue M. Estes, Maurice G. Estes, Dale A. Quattrochi, Sarah N. Hemmings, Virginia G. Wadley, Leslie A. McClure

**Affiliations:** 1 Department of Biostatistics, University of Alabama at Birmingham, Birmingham, Alabama, United States of America; 2 Department of Environmental Health Sciences, University of Alabama at Birmingham, Birmingham, Alabama, United States of America; 3 Universities Space Research Association at NASA Marshall Space Flight Center, Huntsville, Alabama, United States of America; 4 Earth Science Office at NASA Marshall Space Flight Center, Huntsville, Alabama, United States of America; 5 Division of Gerontology, Geriatrics, and Palliative Care, School of Medicine, University of Alabama at Birmingham, Birmingham, Alabama, United States of America; University Hospital La Paz, Spain

## Abstract

Studies of the effect of air pollution on cognitive health are often limited to populations living near cities that have air monitoring stations. Little is known about whether the estimates from such studies can be generalized to the U.S. population, or whether the relationship differs between urban and rural areas. To address these questions, we used a satellite-derived estimate of fine particulate matter (PM_2.5_) concentration to determine whether PM_2.5_ was associated with incident cognitive impairment in a geographically diverse, biracial US cohort of men and women (n = 20,150). A 1-year mean baseline PM_2.5_ concentration was estimated for each participant, and cognitive status at the most recent follow-up was assessed over the telephone using the Six-Item Screener (SIS) in a subsample that was cognitively intact at baseline. Logistic regression was used to determine whether PM_2.5_ was related to the odds of incident cognitive impairment. A 10 µg/m^3^ increase in PM_2.5_ concentration was not reliably associated with an increased odds of incident impairment, after adjusting for temperature, season, incident stroke, and length of follow-up [OR (95% CI): 1.26 (0.97, 1.64)]. The odds ratio was attenuated towards 1 after adding demographic covariates, behavioral factors, and known comorbidities of cognitive impairment. A 10 µg/m^3^ increase in PM_2.5_ concentration was slightly associated with incident impairment in urban areas (1.40 [1.06–1.85]), but this relationship was also attenuated after including additional covariates in the model. Evidence is lacking that the effect of PM_2.5_ on incident cognitive impairment is robust in a heterogeneous US cohort, even in urban areas.

## Introduction

Cognitive decline (CD) is the key criterion for all definitions of dementia including Alzheimer disease (AD) and vascular dementia (VaD) [1]–[4]. Identification of modifiable risk factors for CD can inform public health policy and may one day help to reduce the incidence of dementia.

Deleterious effects of traffic pollution on cognition have been studied in children [5], a small sample of middle-aged adults [6], and the elderly [7], [8]. Some components of traffic pollution make up the more general class of pollutants whose aerodynamic diameter is less than or equal to 2.5 µm (PM_2.5_). This general class of pollutants has been implicated in overall mortality [9], hospital admissions rates for cardiovascular and respiratory diseases [10], and ischemic strokes [11]. Recently, increased exposure to PM_2.5_ has been shown to be equivalent to two years of cognitive aging in older women [12]. While these studies have increased our knowledge of the health effects of PM_2.5_, almost all of them: (1) have studied a specific subpopulation (i.e., children, women or men, or the elderly); (2) were restricted to cities or metropolitan statistical areas (MSAs); or (3) both. To our knowledge, no one has attempted to assess the association between PM_2.5_ and cognitive impairment in a diverse and broadly distributed sample from the U.S. population. Additionally, little is known about whether the relationship between PM_2.5_ and cognitive impairment differs based upon how urban the area is. Previous studies have shown that PM_2.5_ in urban areas, compared to rural areas, contains a larger proportion of black carbon and traffic-related pollution [13], [14]. Concentrations of these specific components, compared to overall PM_2.5_ concentrations, are more strongly and consistently associated with all-cause mortality and cardiovascular outcomes [15]. Therefore, high PM_2.5_ concentrations in urban areas might have a stronger relationship with cognitive impairment than PM_2.5_ in rural areas. However, there has been little investigation of whether “urbanicity” is an effect modifier for the relationship between PM_2.5_ and cognitive impairment.

Previous studies generally have included only participants living in urban areas because PM_2.5_ is difficult to estimate at locations far away from ground-level monitoring stations, which are clustered around cities, such as those maintained by the Environmental Protection Agency. In order to estimate the effects of PM_2.5_ on health at the population level, environmental researchers have investigated alternative methods of measuring PM_2.5_ in cities and in areas with sparse ground-station coverage.

We used a derived estimate of PM_2.5_ concentration that integrated ground-level measurements and Earth-orbiting satellite measurements to investigate the relationship between PM_2.5_ and incident cognitive impairment in a large biracial cohort of men and women spread across urban and rural regions of the coterminous U.S. We further examined whether this relationship differed by urbanicity of residential address.

## Methods

### Ethics Statement

The following Institutional Review Boards (IRBs) of the participating institutions approved the REGARDS study: University of Alabama at Birmingham, University of Vermont, Wake Forest University, and University of Cincinnati. Although the University of Arkansas, Indiana University, and the Medical University of South Carolina (MUSC) participate in the study, the IRBs of these institutions each decided that their respective universities were exempt from obtaining IRB approval, considering their roles. Written informed consent was obtained from all participants.

### Study Sample

Data from participants enrolled in the REasons for Geographic And Racial Differences in Stroke (REGARDS) cohort were used, a cohort of 30,239 participants from the 48 contiguous United States recruited between 2003 and 2007 who continue to be followed up [16]. The primary goal of REGARDS was to understand the reasons that stroke mortality is higher in the stroke buckle (coastal plain of North Carolina, South Carolina, and Georgia) and stroke belt (North Carolina, South Carolina, Georgia, Tennessee, Mississippi, Alabama, Louisiana, and Arkansas) regions of the Southeastern US, compared with the rest of the country, as well as the reasons for the increased stroke mortality in blacks compared to whites. Details of the study are provided elsewhere [16]. In brief, recruitment began by random mailings within region-race-sex strata to inform potential participants of a follow-up telephone call that came two weeks later. Willing participants then participated in a telephone interview to ascertain a myriad of sociodemographic variables and included a medical history. The telephone interview was followed by an in-home visit by a trained health professional to measure physiological characteristics (e.g., systolic blood pressure, weight, height, low-density lipoprotein concentration, among other factors). REGARDS recruited 35% from the stroke belt, 21% from the stroke buckle, and 44% from the rest of the contiguous US. Overall, the cohort is 59% white and 55% female. Our analysis was performed as an assessment of the primary aim of a REGARDS ancillary study, in which the goal was to determine the relationship between PM_2.5_ and incident cognitive impairment.

Of the 30,239 participants initially recruited to REGARDS, we excluded 56 participants due to anomalous data, and 567 were lost to follow-up after the in-home visit, but before the first follow-up telephone call at six months. Six participants were excluded due to missing the date of the initial telephone interview where most participants received their first cognitive assessment. Then, 1,950 participants were excluded due to self-reported history of stroke at baseline, 50 were excluded due to a incident stroke prior to their first cognitive assessment, 2,299 were excluded due to baseline cognitive impairment, 1,436 were excluded due to having only one cognitive assessment, 11 participants were excluded due to missing PM_2.5_ and temperature data, and finally 3,714 participants were excluded for having a SAS geocoding score of 80 or less for the location of their baseline residential address. The exclusion of participants with low geocoding scores decreased the chances for misclassification bias, which is always a concern when using an estimated exposure. These exclusion criteria left a final sample size of n = 20,150.

### Measurement of Outcome (Incident CD)

The large sample size and geographic distribution of the REGARDS participants necessitated a simple cognitive assessment that could be delivered over the telephone. Therefore, the Six-Item Screener (SIS) was used to determine whether a participant was cognitively impaired or intact [17]. Developed as a very brief global measure of cognitive status that could be administered over the telephone, the SIS assesses three-item recall, as well as orientation to year, month, and day of the week [17]. The SIS was administered at baseline to REGARDS participants beginning in December 2003 and continues to be administered annually over the telephone. Possible scores on the SIS ranged from 0–6 correct responses. All participants in the current study had a score of 5 or 6 at baseline (cognitively intact) [17]. Participants were subsequently classified as having incident cognitive impairment if the score from their most recent SIS assessment was 4 or less, and they were classified as intact if their SIS score was 5 or greater. In prior research involving a second-stage clinical evaluation, these cut-off values had a sensitivity of 74% and a specificity of 80% for a combined endpoint of cognitive impairment and dementia diagnoses in a community sample [17]. The SIS has been used to identify cognitively impaired elderly emergency room patients [18], as well as older, depressed patients who were impaired in a large randomized controlled trial [19]. Impaired scores on the SIS have been linked to cardiovascular events [20], cardiovascular risk factors [21], [22], and kidney dysfunction [23].

### Measurement of Exposure (PM_2.5_)

Since about 20% of REGARDS participants were living in rural or mixed urban and rural areas at baseline, the clustering of EPA Air Quality System (AQS) ground-level monitoring stations around cities complicated estimation of the exposure for rural participants. One method for estimating PM_2.5_ over large geographic areas is to use satellite measurements of aerosol optical depth (AOD) from the Moderate Resolution Imaging Spectroradiometer (MODIS) instrument on the National Aeronautics and Space Administration (NASA) Terra and Aqua satellites. AOD is a measure of the reflection of light due to solid, liquid, and gas particles in the air column [24]. Advantages of MODIS measurements include high spatial resolution (10 km) and daily availability of data. However, AOD measurements are not available in the presence of clouds, which in some regions and seasons greatly reduces data coverage. Also, AOD measurements must be used in statistical methods to estimate ground-level PM_2.5_, and the correlation between AOD measurements and AQS measurements can vary across the U.S., or with weather conditions [25].

To leverage the strengths of both AQS and MODIS measurements, we estimated daily PM_2.5_ concentrations for the locations of REGARDS participants from 2003 to 2009, using a method which combined the two data sources using a national version of the Al-Hamdan et al. (2009) algorithm, which has been described in detail elsewhere [24], [26]. We provide a short summary of the approach here.

First, we estimated ground-level PM_2.5_ from MODIS AOD using regression equations per EPA region per season [27]. The regional B-spline smoothing algorithm of Al-Hamdan et al. (2009), which also involved a quality control procedure for the EPA AQS data and a bias adjustment procedure for the MODIS data, was then spatially expanded and used to generate continuous spatial surfaces of daily PM_2.5_ on a 10-km grid for the coterminous U.S. from the combination of AQS PM_2.5_ measurements and the MODIS-estimated PM_2.5_ [26]. The result is an estimated PM_2.5_ surface with less error than the surface estimated using AQS alone [24].

Daily exposures were estimated for the geocoded location of REGARDS participants’ baseline residences enrolled between 2003 and 2007. From these daily estimates of PM_2.5_ concentration, we then estimated the 1-year mean PM_2.5_ concentration, leading up to and including the date of the baseline telephone interview. One year has been established as a sufficient time frame to use to observe effects of PM_2.5_ on incident cognitive impairment in a cohort of older men [8], [12]. Since REGARDS recruitment began in 2003, and our exposure data set began on January 1, 2003, some participants did not have data for 364 days prior to baseline. In those cases, a mean PM_2.5_ concentration was estimated using the available days prior to baseline.

### Measurement of Covariates

#### Temperature

Temperature estimates for each participant’s geocoded location were taken from the North American Land Data Assimilation System (NLDAS), a meteorological ‘re-analysis’ data set produced jointly by NASA, the National Oceanic and Atmospheric Administration (NOAA), and universities to facilitate numerical weather forecasting and analysis. The NLDAS data are derived from the analysis fields of the National Centers for Environmental Prediction (NCEP) North American Regional Reanalysis (NARR), which are at a 32-km spatial resolution and a 3-hourly temporal frequency. The NARR gridded data are temporally disaggregated to the NLDAS hourly frequency and spatially interpolated to the finer 1/8 degree (∼12-km) NLDAS grid. Additionally, the air temperature data are adjusted to account for the vertical difference between the NARR and NLDAS-2 terrain height fields. The details of the spatial interpolation, temporal disaggregations, and vertical adjustment are those employed in LDAS-1 [28]. This data set provides long-term (1979– present) spatially and temporally continuous coverage over a grid spanning the coterminous U.S. and parts of northern Mexico and southern Canada.

For purposes of linking with REGARDS participant data, daily maximum air temperature values for 2003–2009 were defined as the highest hourly NLDAS temperature for each 24-hour period ending at midnight local standard time. The trailing 1-year mean (°C) of the NLDAS daily maximum temperatures for the grid cell containing the geographic location of the participants’ residence was used to define the temperature exposure for each participant.

#### Other covariates

Season was assigned based upon the date of the baseline telephone interview. Age (years), sex, race (black or white), region (stroke buckle, stroke belt, or rest of U.S.), income ($75,000 and above, $35,000–$74,000, $20,000–$34,000, less than $20,000, or refused), education level (college graduate and above, some college, high school graduate, or less than high school), depressive symptoms (4-item CES-D scale), smoking status (current, past, or never), alcohol use (heavy [7 or more drinks/week for women, 14 or more drinks/week for men], moderate [more than 0, but less than 7 drinks/week for women, more than 0, but less than 14 drinks/week for men], or none), and level of exercise (4 or more times/week, 1–3 times/week, or none) were assessed during the baseline telephone interview, and as such were all self-reported variables. Participants were classified as depressed if their score on the 4-item CES-D scale was 4 or greater (range 0–12) [29].

An in home visit followed the telephone interview, where a trained health professional drew blood, collected urine, and measured physiological factors including blood pressure (mmHg, the average of two seated values), lipid levels (mg/dL), glucose levels (mg/dL), height, and weight. Participants were classified as having dyslipidemia if their total cholesterol was greater than or equal to 240 mg/dL, low-density lipoprotein (LDL) was greater than or equal to 160 mg/dL, high-density lipoprotein (HDL) was less than or equal to 40 mg/dL, or they used a cholesterol medication. Participants were classified as hypertensive if their systolic blood pressure (SBP) was greater than or equal to 140 mmHg, diastolic blood pressure (DBP) was greater than 90 mmHg, or the participants self-reported taking anti-hypertensive medications. Participants were classified as diabetic if their fasting glucose was greater than or equal to 126 mg/dL, non-fasting glucose was greater than 200 mg/dL, or they self-reported medication or insulin use. Body mass index (kg/m^2^) was estimated using measured weight and height. Telephone interviews at 6-month intervals were used to ascertain incident strokes, which were then adjudicated by a team of neurologist stroke experts [16].

### Statistical Methods

Logistic regression was chosen for modeling the outcome (incident cognitive impairment at the most recently observed assessment). Although survival analysis was considered, we could not verify the necessary assumptions that: (1) the survival function was strictly non-increasing; or (2) the censoring was noninformative, because participants with low cognitive function have a tendency to drop out of long-term studies (i.e., greater than 1 year of follow-up) [30]. The loss of power from using logistic regression was less significant than the potential biases that could have resulted from assumption violations if survival analysis had been used. Additionally, the potential noise in a brief measure of cognitive impairment with a restricted score range might have made selection of an appropriate covariance structure difficult, and thus misspecification more likely, had generalized estimating equations (GEE) been used.

Four logistic regression models were fitted to determine whether there was an association between the odds of incident cognitive impairment and PM_2.5_. These models were selected *a priori* to ascertain the robustness of the estimated relationship between cognitive impairment and PM_2.5_, as more and more potential confounders were added to the model. Model 1 included PM_2.5_ (µg/m^3^), length of follow up (days between baseline telephone interview and most recent cognitive assessment), and the potential confounders temperature, season, and incident stroke [4], [11]. Model 2 included the factors in model 1, and added the following demographic factors: age, race, region, education, and income. Model 3 included all the factors in model 2, and added behavioral factors: smoking status, alcohol use, exercise, and BMI. Finally, model 4 included all factors in model 3, and added known comorbidities of cognitive impairment: presence of depressive symptoms, dyslipidemia (present or absent), diabetes (present or absent), and hypertension (present or absent). An odds ratio and approximate large sample Wald’s 95% confidence interval were estimated for a 10 µg/m^3^ increment of PM_2.5_ for each model. Only participants with values for all covariates were included in each model fit. This procedure resulted in 0%, 0.06%, 4.0%, and 9.4% missing data for each model, respectively. In the final model, most of the missingness was caused by missing values for diabetes and dyslipidemia, which integrated information from the telephone interview, in-home visit, and laboratory analysis of blood and urine. This level of missingness did not warrant more sophisticated forms of accounting for missingness (e.g., multiple imputation).

To determine whether level of urbanicity was an effect modifier of a potential relationship between PM_2.5_ and incident cognitive impairment, both the main effect for urbanicity and the interaction between urbanicity and PM_2.5_ were added to the models, and the models were re-fitted. Urbanicity was defined as the proportion of urban area in the participant’s US census tract according to the 2000 US census: ≤25% was rural, 25%–75% was mixed, and ≥75% was urban. Odds ratios and approximate large-sample Wald’s 95% confidence intervals were estimated for a 10 µg/m^3^ increase in PM_2.5_ within each level of urbanicity, for all four models.

A *post hoc* sensitivity analysis was conducted, where cognitive impairment was defined as being impaired on the *two* most recent SIS assessments. Four logistic regression models identical to the four models in the main analysis were fitted, and the results were reported as the estimated odds ratios for a 10 µg/m^3^ increase in PM_2.5_ and large-sample Wald’s approximate 95% confidence intervals.

Data set creation was done using SAS version 9.2 (Cary, NC). All statistical procedures were performed using SAS version 9.3 (Cary, NC). The code files that created the final data set (Code S1), computed descriptive statistics (Code S2), and performed the statistical modeling (Code S3) are available as supporting information.

## Results

Of the 20,150 participants, 3,408 had less than 364 days of exposure data available prior to baseline because they were recruited during 2003. These participants had a mean of 238 days (sd = 73) of data (minimum of 25, maximum of 352). Therefore, 17% of the participants had estimates of mean PM_2.5_ concentration that may have been slightly less stable than the rest of the sample.

Table 1, Table 2, Table 3, Table 4 show the summary statistics for each group of covariates by quantile of PM_2.5_ exposure. We chose to present the summary statistics in this manner because, in the main analysis, we were interested in which covariates might affect the estimated relationship between PM_2.5_ and incident cognitive impairment. In order for a covariate to have such an effect, the covariate must have a relationship with PM_2.5_. Most categories for each covariate did not show a monotonic increase or decrease in proportion of participants in each category of increasing PM_2.5_ exposure, indicating that linear relationships between those covariates and PM_2.5_ exposure were unlikely in our sample. One exception was race, where blacks had a 20 percentage point increase in proportion of participants between the lowest and highest quantiles of PM_2.5_ exposure. Additionally, there was a 24 percentage point increase in the proportion of participants living in the Stroke Belt from the lowest quantile to the highest quantile, coupled with a 24 percentage point decrease in the proportion of participants living in the rest of the US over the same quantiles, although these changes were not strictly monotonic.

**Table 1:** Summary statistics of follow-up time and known confounders by PM2.5 (µg/m^3^) quantiles.

		Quartile of PM_2.5_			
Variable	Level	6.6–12.2	12.2–13.6	13.6–14.8	14.8–21.0
Temperature (C),Mean(SD)		17.4(5.0)	17.9(3.5)	15.9(3.4)	16.2(3.1)
Follow-up (days),Mean(SD)		1675(736)	1672(674)	1682(662)	1772(649)
Season, %					
	Spring	29.9	24.9	27.9	18.5
	Summer	29.1	30.2	27.3	29.5
	Fall	18.1	22.4	20.7	26.7
	Winter	23.0	22.5	24.1	25.3
Incident stroke, %		1.9	1.8	2.1	1.9

**Table 2:** Summary statistics of demographics by PM2.5 (µg/m^3^) quantiles.

		Quartile of PM_2.5_			
Variable	Level	6.6–12.2	12.2–13.6	13.6–14.8	14.8–21.0
Age (years), Mean(SD)		64.8(9.2)	64.2(9.3)	64.1(9.2)	64.0(9.2)
Race, %					
	Black	29.1	36.8	43.0	49.0
	White	70.9	63.2	57.1	51.0
Male gender, %		48.0	42.2	43.4	41.4
Education, %					
	College graduate and above	42.8	35.3	36.4	37.5
	Some college	26.9	26.7	26.2	28.9
	High school graduate	22.7	26.0	26.6	24.4
	Less than high school	7.6	12.0	10.7	9.2
Stroke region, %					
	Belt	20.4	35.9	35.6	44.7
	Buckle	11.4	36.8	21.8	9.0
	Rest of US	68.2	27.3	42.7	46.4
Income, %					
	≥$75,000	20.5	16.4	17.0	17.7
	$35,000–$74,000	33.0	30.4	30.9	32.3
	$20,000–$34,000	21.8	23.3	23.4	24.1
	< $20,000	13.4	17.3	16.9	14.6
	Refused	11.3	12.7	11.8	11.2

**Table 3:** Summary statistics of behavioral factors by PM2.5 (µg/m^3^) quantiles.

		Quartile of PM_2.5_			
Variable	Level	6.6–12.2	12.2–13.6	13.6–14.8	14.8–21.0
BMI (kg/m^2^), Mean(SD)		29.2(6.1)	29.3(6.2)	29.4(6.3)	29.4(6.2)
Smoking status, %					
	Current	11.9	13.5	14.4	14.8
	Past	42.4	39.1	40.6	37.5
	Never	45.7	47.5	45.1	47.7
Alcohol use, %					
	Heavy	5.3	4.3	3.7	3.5
	Moderate	40.7	32.3	33.3	33.7
	None	54.0	63.5	63.0	62.8
Exercise, %					
	4 or more times/week	31.4	30.6	28.4	28.1
	1–3 times/week	37.4	36.6	38.2	39.0
	None	31.2	32.9	33.4	32.9

**Table 4:** Summary statistics of known comorbidities by PM2.5 (µg/m^3^) quantiles.

	Quartile of PM_2.5_			
Variable	6.6–12.2	12.2–13.6	13.6–14.8	14.8–21.0
Diabetic, %	17.6	21.2	21.5	19.7
Hypertensive, %	53.2	58.2	58.6	59.3
Dyslipidemia, %	57.9	59.4	58.2	57.7
Depressed, %	8.2	10.6	9.7	9.4

Of the total sample (n = 20,150), 1,633 participants (8%) were classified as cognitively impaired at their most recent follow-up. Table 5 shows the odds ratios and approximate 95% Wald’s confidence intervals for a 10 µg/m^3^ change in PM_2.5_ for each of the four models, both for the main analysis (impaired on the most recent assessment) and sensitivity analysis (impaired on both of the two most recent assessments). Model 1 indicated that there was no association between PM_2.5_ and the odds of incident cognitive impairment. The odds ratio decreased sharply after demographics were added (model 2), with an odds ratio close to 1. This estimated odds ratio was more or less constant for models 3 and 4. We have provided the estimated odds ratios and confidence intervals for model 4 as supplementary material for interested readers (Table S1).

**Table 5:** Odds ratios (95% confidence intervals) measuring the effect of a 10 µg/m^3^ increase in PM_2.5_ on odds of incident cognitive impairment.

Model	Main analysis (n = 20,150)	Sensitivity analysis (n = 18,180)
1	1.26 (0.97–1.64)	1.02 (0.61–1.70)
2	1.02 (0.76–1.37)	0.75 (0.42–1.33)
3	0.97 (0.72–1.31)	0.72 (0.39–1.30)
4	0.98 (0.72–1.34)	0.71 (0.38–1.32)

Removal of the participants who did not have 365 days of exposure data changed inference for model 1, with the effect of a 10 µg/m^3^ increase in PM_2.5_ concentration now significantly associated with the odds of incident cognitive impairment [OR (95% CI): 1.46 (1.08–1.98)]. However, the effect of PM_2.5_ remained non-significant in models 2 through 4.

The models investigating effect modification by urbanicity did not show strong evidence that the effect of PM_2.5_ on incident cognitive impairment differed among rural, mixed, or urban areas. Table 6 shows the distribution of participants in each level of urbanicity (rural, mixed, or urban) across the same quantiles of PM_2.5_ that were used in Table 1, Table 2, Table 3, Table 4. The proportions of participants across quantiles did not change drastically for the rural, mixed, or urban areas. Table 7 presents the odds ratios and 95% confidence intervals for a 10 µg/m^3^ increase in PM_2.5_ exposure, separated by urbanicity for models 1–4. The effect of PM_2.5_ for urban areas in model 1 was significant (OR [95% confidence interval]: 1.40 [1.06–1.85]), but this relationship was attenuated towards 1 in models 2–4. The effect of PM_2.5_ for mixed areas in models 2 and 3 were significant (OR [95% confidence intervals]: 0.33 [0.11–0.99] and 0.32 [0.11–0.98], respectively), but the point estimates did not vary much across the models, indicating potentially spurious significant results. Table 8 presents the distribution of race by urbanicity, which shows that the distribution of black participants is more skewed towards urban areas, compared to the distribution of whites over the different levels of urbanicity. Differences of exposure between the races, which were alluded to in the paragraph above, might explain the attenuation of the odds ratio for the interaction between PM_2.5_ and urbanicity. Additionally, Table 9 shows that the proportion of participants living in urban areas appears higher in the rest of the US compared to the Stroke Belt and Stroke Buckle, which might also explain the attenuation of the interaction between PM_2.5_ and urbanicity for similar reasons.

**Table 6:** Distribution of urbanicity by PM2.5 (µg/m^3^) quantiles.

		Quartile of PM_2.5_			
Urbanicity^1^,%	Level	6.6–12.2	12.2–13.6	13.6–14.8	14.8–21.0
	Rural	9.2	13.3	11.1	7.1
	Mixed	9.0	14.1	11.1	8.2
	Urban	81.9	72.6	77.9	84.8

1 Rural is ≤25% urban, mixed is 25%–75% urban, and urban is ≥75% urban.

**Table 7:** Odds ratios (95% confidence intervals) measuring the effect of a 10 µg/m^3^ increase in PM_2.5_ on odds of incident cognitive impairment, by urbanicity group.

	Urbanicity group		
Model	Rural	Mixed	Urban
1	0.68 (0.23–2.08)	0.41 (0.14–1.16)	1.40 (1.06–1.85)
2	0.61 (0.19–1.92)	0.33 (0.11–0.99)	1.13 (0.83–1.54)
3	0.60 (0.19–1.93)	0.32 (0.11–0.98)	1.07 (0.78–1.47)
4	0.79 (0.23–2.68)	0.34 (0.11–1.04)	1.06 (0.77–1.48)

**Table 8:** Distribution of race by urbanicity.

	Urbanicity category		
Race, %	Rural (n = 2,046)	Mixed (n = 2,128)	Urban (n = 15,976)
Black	3.3	5.0	91.7
White	14.2	14.6	71.2

**Table 9:** Distribution of stroke region by urbanicity.

	Urbanicity category		
Region, %	Rural (n = 2,046)	Mixed (n = 2,128)	Urban (n = 15,976)
Belt	12.6	12.5	74.9
Buckle	17.9	20.4	61.7
Rest of US	5.1	4.9	90.0

The sensitivity analysis with the modified definition of incident impairment included n = 18,180 participants, 399 (2%) of whom were impaired on both of the previous two assessments. The results of this analysis are also reported in Table 5. Each of the odds ratios was not reliably different from 1.

## Discussion

When accounting for potential confounders (temperature, season, and incident stroke), the odds of incident impairment for a 10 µg/m^3^ increase in PM_2.5_ concentration increased by 26%; however, the 95% confidence interval for this estimate did cross 1, indicating a lack of strong evidence for an effect of increased particulate matter concentration on incident cognitive impairment in a biracial, bigender national cohort of U.S. adults at least 45 years of age. Sensitivity analyses removing those with <365 days of exposure indicated that the effect of PM_2.5_ was significantly associated with incident cognitive impairment after adjusting for potential confounders. However, after additional adjustment, the association was no longer significant.

Because of the diverse cohort included in these analyses, conditioning upon relevant demographic variables (age, race, gender, region of U.S. residence, income, and education level) was prudent in subsequent models to account for differences in impairment across strata. These covariates were included in model 2, which resulted in attenuation of the odds ratio to 1.02. Such a large decrease in the estimated odds ratio for PM_2.5_ from model 1 to model 2 indicates that at least one of the demographic covariates was related to PM_2.5_ concentration. The data presented in Table 2 suggest that blacks were exposed to higher levels of PM_2.5_ than whites, and that participants living in the Stroke Belt showed a stronger tendency to have higher levels of PM_2.5_ exposure compared to participants living in other parts of the country. No other covariates in the study (demographic or otherwise) displayed such clear linear relationships with PM_2.5_ concentration. Because a larger proportion of black participants were classified as impaired compared to whites (11.2% versus 6.1%, respectively), race might be a critical confounder to account for when estimating the relationship between PM_2.5_ and cognitive impairment. Additionally, elevated incidence of cognitive impairment in the Stroke Belt has already been established [31], and so area of residence in the US might be another important confounder of the relationship between PM_2.5_ and incident cognitive impairment. Establishment of these two variables as potential confounders accomplishes one important goal of this study, which was to see if the relationship between PM_2.5_ and incident cognitive impairment was affected by any of a number of covariates in a large biracial national cohort.

Table 7 shows that there was moderate indication that a 10 µg/m^3^ increase in PM_2.5_ exposure was associated with the odds of incident cognitive impairment for urban participants, but this relationship was attenuated in all subsequent models. Thus, we find little evidence that urbanicity is an effect modifier for the relationship between PM_2.5_ and incident cognitive impairment. Previous studies have included a proxy for urbanicity of residential address, such as distance of residence to nearest busy road, but we know of no other study that has investigated the potential effect modification of urbanicity on the relationship between PM_2.5_ and incident cognitive impairment based upon U.S. census data.

The sensitivity analysis in which cognitive impairment was defined based on the two most recently observed assessments provided null results for the relationship between PM_2.5_ and incident cognitive impairment. We conducted the sensitivity analysis because of the imperfect nature of the SIS, reasoning that participants who had been impaired on the two most recent assessments were likely experiencing enduring impairment, thus removing some of the noise in the outcome measurement. This approach did not reveal a previously undiscovered relationship, providing further evidence to support the null results found in the main analysis.

A causal relationship between PM_2.5_ and cognitive impairment has been implied in much of the literature. Experimental evidence in mice has shown that increased exposure to air pollutants results in an increased expression of biomarkers for inflammation [32], and observational studies of children, adults, and canines residing in large Mexican cities have shown similar increases in biomarkers of inflammation, the same biomarkers associated with the hallmarks of Alzheimer disease [33]–[36].

However, we were not able to detect a significant association between PM_2.5_ and incident cognitive impairment in our cohort, and urbanicity showed no evidence of being an effect modifier for this relationship. Most of the previous studies in this area have used populations that were older and less diverse, by both race and gender, than our cohort. An exception was the study using a subsample of NHANES-III that was diverse both by race and by gender, which found that the association between PM_10_ and cognitive impairment disappeared after the addition of demographic variables to the model [6]. We speculate that cognitive impairment has a range of risk factors (e.g., stroke), and that some of those risk factors might be more or less important for becoming impaired depending upon the subpopulation of the coterminous U.S. under consideration. In a diverse population, the potential effect of PM_2.5_ on cognitive impairment might not be seen because other risk factors for impairment might be important.

### Study Strengths

The strengths of this study include the use of a nationwide, demographically diverse U.S. cohort compared to previous studies on air pollution and cognitive decline that were conducted in urban areas [8], [12], [37]. We used measurements of the exposure and the outcome that might have created greater misclassification bias compared to these previous studies, in order to reduce the selection bias that was potentially present in those same previous studies. This trade-off led to the discovery that race might be a critical confounder of the relationship between PM_2.5_ and incident cognitive impairment. Because of this confounder, results from studies of air pollution and cognitive decline that have less diverse samples might not be generalizable to some parts of the U.S. population. Specifically, when we accounted for demographics in model two, which included race, we found little evidence that a relationship between increased levels of PM_2.5_ and incident cognitive impairment exists. We speculate that race might be an important confounder when studying other conditions potentially affected by PM_2.5_ that have known differences in incidence by race (e.g., coronary heart disease or stroke).

### Study Limitations

Compared to previous studies concerning air pollution and cognitive decline, our study used potentially noisier measures for both the outcome and the exposure. The SIS was designed as a simple screening tool that could be delivered over the telephone and is not equivalent to a physician’s diagnosis of a cognitive disorder (e.g., dementia). Even so, impairment classification derived from the SIS has been shown to agree much of the time with physician diagnoses [17]. Furthermore, incident impairment as defined in the present study has been found to be higher in participants with older age, lower education, black race, residence in the Stroke Belt [31], and higher Framingham Stroke Risk Profile scores [38], lending further support for its overall validity and utility in a large cohort with a wide geographic distribution, such as REGARDS. Using such a measure was necessary given the large sample size of the REGARDS study and the geographic distribution of its participants.

Given that 10% of REGARDS participants lived in census tracts that were between 25% and 75% urban, and 10% of the REGARDS participants lived in census tracts that were less than 25% urban, it was necessary to utilize estimates of PM_2.5_ that were more reasonable than a measurement from an AQS station that might have been a substantial distance from the participant’s residential address. Therefore we used a satellite-derived estimate of PM_2.5_ concentration, which integrated AOD data with the AQS data. Even after quality control on the AQS measurements and bias-correction procedures on the satellite-derived estimates of PM_2.5_ concentration, a derived estimate for a rural participant was not going to be as reliable as an AQS estimate for an urban participant. From cross-validation procedures, however, we reasoned that the derived estimate at a rural participant’s location was more reliable than an AQS measurement from a station potentially far away from the participant’s residential address. Additionally, the correlation between AOD estimates is known to be poor in the Southwestern part of the U.S., as well as in cooler months [24], [26], [39], [40]. Few REGARDS participants live in the Southwestern U.S., so systematic misclassification bias because of the poor correlation in the Southwestern U.S. should have been minimal.

Additionally, use of a derived estimate of air pollution exposure in a health model can induce a Berkson error, because a derived exposure that is known to have measurement error is used as if it did not have measurement error when included in the health model [41]. Depending on the smoothness of the estimated surface, the bias of parameter estimates using a logistic regression model can range from −0.55 to −6.72. The authors concede that this bias is possible in our sample, and further investigation of its potential existence should be pursued in future analyses. However, all studies that use a derived measurement are subject to this potential bias, so our results should be no more affected than previous studies.

Finally, the 1-year exposure interval might not have been the ideal interval to observe the association between PM_2.5_ and incident cognitive impairment. Previous studies have observed similar effects on cognition within this time frame in elderly populations [8], [12], indicating that one year may be a sufficiently long time frame, but not necessarily ideal. Further investigation of the ideal time frame to use for studies of PM_2.5_ and cognitive impairment would be valuable.

## Conclusions

Our study has not shown a significant effect of a 1-year average PM_2.5_ exposure on the odds of incident cognitive impairment. The effect was also non-significant when cognitive impairment was defined by the two most recent assessments. This relationship was not modified by the urbanicity of the participant’s residential address, indicating no evidence that particulate matter has a stronger/weaker association with cognitive impairment in rural, mixed, or urban areas. This study was the first attempt to estimate the association between PM_2.5_ and incident cognitive impairment using a large biracial cohort of men and women spread across urban and rural regions of the coterminous U.S.

## Supporting Information

Table S1 **Odds ratios and 95% Wald confidence intervals for incident cognitive impairment, model 4, all covariates.**
(DOC)

Code S1 **Code for data merge.** SAS program file detailing the merging of source data files to produce the final data set.
(PDF)

Code S2 **Code for descriptive statistics.** SAS program file detailing the descriptive statistics calculated on the final data set.
(PDF)

Code S3 **Code for analyses.** SAS program file detailing the analyses for the study, using logistic regression.
(PDF)
