# Correction: Fine Particulate Matter and Incident Cognitive Impairment in the REasons for Geographic and Racial Differences in Stroke (REGARDS) Cohort

**DOI:** 10.1371/journal.pone.0125137

**Published:** 2015-04-17

**Authors:** Matthew Shane Loop, Shia T. Kent, Mohammad Z. Al-Hamdan, William L. Crosson, Sue M. Estes, Maurice G. Estes, Dale A. Quattrochi, Sarah N. Hemmings, Virginia G. Wadley, Leslie A. McClure

There is an error in the second paragraph of the “Temperature” portion of the “Measurement of Covariates” subsection of the Methods. The correct paragraph is: For purposes of linking with REGARDS participant data, daily mean air temperature values for 2003–2009 were defined as the average of the hourly values for a 24-hour period ending at midnight local standard time. The trailing 1-year mean (°C) of the NLDAS daily mean temperatures for the grid cell containing the geographic location of the participants’ residence was used to define the temperature exposure for each participant.
