# Supplementary material for: Fine Particulate Matter and Incident Cognitive Impairment in the REasons for Geographic and Racial Differences in Stroke (REGARDS) Cohort
Source: PLoS One. 2013 Sep 25;8(9):e75001. doi: 10.1371/journal.pone.0075001 (PMC3783452; doi:10.1371/journal.pone.0075001)
Supplement: Table S1 — Odds ratios and 95% Wald confidence intervals for incident cognitive impairment, model 4, all covariates. (DOC) [file pone.0075001.s001.doc]

**Table S4. Odds ratios and 95% Wald confidence intervals for incident cognitive impairment, model 4, all covariates.**

| **Variable** |  | **OR (95% CI)** |
| --- | --- | --- |
| PM_2.5_, 10 µg/m^3^ |  | 0.98 (0.72 – 1.34) |
| Season |  |  |
|  | Fall vs. Winter | 0.97 (0.83 – 1.14) |
|  | Spring vs. Winter | 1.01 (0.86 – 1.17) |
|  | Summer vs. Winter | 0.95 (0.81 – 1.10) |
| Gender |  |  |
|  | Female vs. Male | 0.61 (0.54 – 0.68) |
| Race |  |  |
|  | Black vs. White | 1.69 (1.49 – 1.91) |
| Region |  |  |
|  | Stroke Belt vs. rest of US | 0.96 (0.83 – 1.11) |
|  | Stroke Buckle vs. rest of US | 0.93 (0.79 – 1.10) |
| Education |  |  |
|  | College graduate and above vs. some college | 0.98 (0.84 – 1.15) |
|  | High school graduate vs. some college | 1.21 (1.04 – 1.41) |
|  | Less than high school vs. some college | 2.07 (1.74 – 2.48) |
| Income |  |  |
|  | $20,000 - $34,000 vs. less than $20,000 | 0.81 (0.69 – 0.95) |
|  | $35,000 - $74,000 vs. less than $20,000 | 0.59 (0.49 – 0.70) |
|  | $75,000 and above vs. less than $20,000 | 0.45 (0.36 – 0.57) |
|  | Refused vs. less than $20,000 | 0.85 (0.70 – 1.04) |
| Smoking status |  |  |
|  | Current vs. Past | 0.65 (0.55 – 0.78) |
|  | Never vs. Past | 0.95 (0.84 – 1.07) |
| Alcohol use |  |  |
|  | Heavy vs. None | 0.76 (0.55 – 1.05) |
|  | Moderate vs. None | 0.87 (0.77 – 0.99) |
| Exercise level |  |  |
|  | 1-3 times/week vs. None | 0.94 (0.82 – 1.07) |
|  | 4 or more times/week vs. None | 0.99 (0.86 – 1.14) |
| Diabetes |  |  |
|  | Absent vs. Present | 0.85 (0.74 – 0.97) |
| Hypertension |  |  |
|  | Absent vs. Present | 0.79 (0.70 – 0.89) |
| Dyslipidemia |  |  |
|  | Absent vs. Present | 0.99 (0.88 – 1.11) |
