# Supplementary material for: Fine Particulate Matter and Incident Cognitive Impairment in the REasons for Geographic and Racial Differences in Stroke (REGARDS) Cohort
Source: PLoS One. 2013 Sep 25;8(9):e75001. doi: 10.1371/journal.pone.0075001 (PMC3783452; doi:10.1371/journal.pone.0075001)
Supplement: Code S1 — Code for data merge. SAS program file detailing the merging of source data files to produce the final data set. (PDF) [file pone.0075001.s002.pdf]

```
********************************************************
Author: Matthew Shane Loop

Purpose: This program will re-merge the pm
	and cognition data for the project. Details about
	the project can be found on the Regards and Environment
	Wiki page on the SSG Wiki.
********************************************************;
LIBNAME REGARDS "..\..\Data\regards\20110401";
LIBNAME PM "..\..\Data\pm";
LIBNAME TEMP "..\..\Data\temperature";
LIBNAME BASE "..\..\Data\regards\baseline";
LIBNAME FINAL "..\data";

***************************
Import the relevant REGARDS data and perform the following exclusion criteria:
* Exclude those who are not in the cognitive data set (lost to follow-up from
baseline)
* Exclude those with a missing date for the initial telephone interview
* Exclude those with a baseline self-reported stroke
* Excluded those with an incident stroke prior to the first SIS assessment
* Exclude those with baseline cognitive impairment
* Exclude those with only 1 SIS assessment
* Exclude those with geo_scores of 80 or less
*****************************;

* Import the relevant REGARDS data ;
DATA CALCVARS;
	SET REGARDS.CALCVARS;
	KEEP ID_NUM INTDATE BMI RACE GENDER ED_CAT REGION INCOME_4CAT
SMOKE Alc_NIAAA Exercise_cat Lipidemia_meds_labs Diab_SRMed_glu
Hyper_SRmeds_bp STROKE_SR AGE URBANGRP CESD;
RUN; * Read in 30,183 observations;
DATA COG;
	SET REGARDS.COGNITIVE_SIS;
	KEEP STROKE1 ID_NUM INCIDENT_IMPAIRMENT INCIDENT_STROKE
INCIDENT_IMPAIRMENT2 TOTAL_ASSESS BASELINE_IMPAIR ASSESS_INTERVAL;
RUN; * Read in 29,616 observations;
DATA GEO;
	SET BASE.SAS_GEOCODED;
	KEEP ID_NUM GEO_SCORE;
RUN;

* Make ID_NUM a numeric variable in all of these data sets;

		DATA CALC_NUM;
```

```
			SET CALCVARS;
			ID = ID_NUM+0;
			DROP ID_NUM;
		RUN;
		DATA CALCVARS;
			SET CALC_NUM;
			ID_NUM = ID;
			DROP ID;
		RUN;
		DATA COG_NUM;
			SET COG;
			ID = ID_NUM+0;
			DROP ID_NUM;
		RUN;
		DATA COG;
			SET COG_NUM;
			ID_NUM = ID;
			DROP ID;
		RUN;
		DATA GEO_NUM;
			SET GEO;
			ID = ID_NUM+0;
			DROP ID_NUM;
		RUN;
		DATA GEO;
			SET GEO_NUM;
			ID_NUM = ID;
			DROP ID;
		RUN;

* Merge calcvars and cog by id_num;
DATA CALC_COG;
	MERGE CALCVARS(IN=A) COG(IN=B);
	BY ID_NUM;
	IF A AND B;
RUN;		* Read in 29,616. 567 lost to follow-up.;

* Exclude those with a missing date for the initial telephone interview;
DATA HAVEDATE;
	SET CALC_COG;
	IF INTDATE NE .;
RUN;  * Read in 29,610 observations. 6 excluded.;

* Exclude those with a baseline self-reported stroke or missing data for
baseline self-reported stroke;
DATA NOSTROKE;
```

```
	SET HAVEDATE;
	IF STROKE_SR = 'N';
RUN;	* Read in 27,660 observations. 1,950 excluded: 97 excluded for missing,
1,853 excluded for baseline stroke.;

* Exclude those with an incident stroke prior to the first SIS assessment ;
DATA NOINCSTROKE;
	SET NOSTROKE;
	IF INCIDENT_STROKE NE 1;
RUN;		* Read in 27,610 observations. 50 excluded.		;

* Exclude those with baseline cognitive impairment;
DATA INTACT;
	SET NOINCSTROKE;
	IF BASELINE_IMPAIR = 0;
RUN;			* Read in 25,311. 2,299 excluded;

* Exclude those with only 1 SIS assessment;
DATA MULTASSESS;
	SET INTACT;
	IF TOTAL_ASSESS > 1;
RUN;			* Read in 23,875 observations. 1,436 excluded.;

DATA CALC_COG_GEO;
	MERGE MULTASSESS(IN=A) GEO(IN=B);
	BY ID_NUM;
	IF A AND B;
RUN;


****************************
Now, assign the temperature and PM2.5 exposures, which are the
average 1 year exposures leading up to and including the
date of the in-home visit.
*******************************;

* Assign PM2.5 exposures;
DATA REGARDS_DAY;
	SET CALC_COG_GEO;
	DATE1 = '31Dec2002'd;
	DAY = INTDATE - DATE1;
RUN;
DATA PM;
	SET PM.PM_ALL;
RUN;
DATA pm_all_num;
```

```
	SET pm;
	ARRAY days{*} _numeric_;    * Makes a numeric variable for each numeric
variable in the data set, then puts them in an array;
	ARRAY day{3287} _1-_3287; * Creates an array that has all of the days. The
ID variable was created as _0 in the _numeric_ function.;
	DO i=1 TO 3287;
day{i}=days{i+1}; * Makes the value for, say, 03_1 into _1;
	END;
	KEEP ID_NUM _1-_3287;
RUN;
DATA REGARDS_PM;
	MERGE REGARDS_DAY(IN=A) PM_all_num(IN=B);
	BY ID_NUM;
	IF A AND B;
RUN;				* Read in 23,864 observations. 11 excluded.;

DATA PM_AVG;
	SET REGARDS_PM;
	ARRAY MEANS{3287} _1 - _3287;
	PM25=0;
	IF DAY < 365 THEN DO I=1 TO DAY;
		PM25 = MEANS{DAY-I+1}+PM25;
	END;
	ELSE DO K = 1 TO 365;
		PM25 = MEANS{DAY-K+1} + PM25;
	END;
	IF DAY < 365 THEN PM25_AVG = PM25/(DAY);
	ELSE PM25_AVG = PM25/365;
	DROP K I _1 - _3287 DATE1 PM25;
RUN;


* Assign temperature exposures;
DATA TEMP;
	SET TEMP.MEAN_TEMP_ALL;
RUN;
DATA TEMP_all_num;
	SET TEMP;
	ARRAY days{*} _numeric_;    * Makes a numeric variable for each numeric
variable in the data set, then puts them in an array;
	ARRAY day{3287} _1-_3287; * Creates an array that has all of the days. The
ID variable was created as _0 in the _numeric_ function.;
	DO i=1 TO 3287;
day{i}=days{i+1}; * Makes the value for, say, 03_1 into _1;
	END;
	KEEP ID_NUM _1-_3287;
```

```
RUN;

DATA REGARDS_TEMP;
	MERGE REGARDS_DAY(IN=A) TEMP_all_num(IN=B);
	BY ID_NUM;
	IF A AND B;
RUN;

DATA TEMP_AVG;
	SET REGARDS_TEMP;
	ARRAY MEANS{3287} _1 - _3287;
	TEMP=0;
	IF DAY < 365 THEN DO I=1 TO DAY;
		TEMP = MEANS{DAY-I+1}+TEMP;
	END;
	ELSE DO K = 1 TO 365;
		TEMP = MEANS{DAY-K+1} + TEMP;
	END;
	IF DAY < 365 THEN TEMP_AVG = TEMP/(DAY);
	ELSE TEMP_AVG = TEMP/365;
	DROP K I _1 - _3287 DATE1 TEMP;
RUN;

* Merge the two data sets;
DATA PM_COG;
	MERGE PM_AVG(IN=A) TEMP_AVG(IN=B);
	BY ID_NUM;
	IF A AND B;
	MONTH=MONTH(INTDATE);
	IF MONTH=4 OR MONTH=5 OR MONTH=6 THEN SEASON="Spring";
	IF MONTH=7 OR MONTH=8 OR MONTH=9 THEN SEASON="Summer";
	IF MONTH=10 OR MONTH=11 OR MONTH=12 THEN SEASON="Fall";
	IF MONTH=1 OR MONTH=2 OR MONTH=3 THEN SEASON="Winter";
	LAST_COGDATE = INTDATE+ASSESS_INTERVAL;
	FORMAT LAST_COGDATE DATE9.;
	IF GEO_SCORE > 80;
	IF CESD GE 4 THEN DEPRESSED = 1;
	IF CESD < 4 AND CESD NE . THEN DEPRESSED = 0;
	IF CESD = . THEN DEPRESSED = .;
	TEMP_AVG_C = TEMP_AVG - 272.15;
RUN;				* Read in 20,150 observations. Excluded 3,714.;
DATA FINAL.PM_COG;
	SET PM_COG;
RUN;
```
