# Supplementary material for: Fine Particulate Matter and Incident Cognitive Impairment in the REasons for Geographic and Racial Differences in Stroke (REGARDS) Cohort
Source: PLoS One. 2013 Sep 25;8(9):e75001. doi: 10.1371/journal.pone.0075001 (PMC3783452; doi:10.1371/journal.pone.0075001)
Supplement: Code S2 — Code for descriptive statistics. SAS program file detailing the descriptive statistics calculated on the final data set. (PDF) [file pone.0075001.s003.pdf]

```
********************************************************
Author: Matthew Shane Loop

Purpose: This program will re-calculate the descriptive
                    statistics for the pm and cognition data for the project. Details about
      the project can be found on the Regards and Environment
      Wiki page on the SSG Wiki.
*******************************************************;
LIBNAME FINAL "..\data";
DATA PM_COG;
         SET FINAL.PM_COG;
RUN;
PROC UNIVARIATE DATA=PM_COG;
         VAR PM25_AVG;
         HISTOGRAM;
         TITLE "Distribution of PM2.5 values";
RUN;
TITLE;
PROC UNIVARIATE DATA=PM_COG;
         VAR TOTAL_ASSESS;
RUN;

******************************************
Split the PM25_AVG values into quartiles (lowest, second, third, highest),
to mirror less than .25, .25 - .5, .5 - .75, and greater than .75.

Then, we will compute the summary statistics according to each group
of PM2.5 exposures.
********************************************;
DATA PM_COG_CAT;
         SET PM_COG;
         IF PM25_AVG LE 12.17288 THEN PM_Q = "lowest";
         IF PM25_AVG > 12.17288 AND PM25_AVG LE 13.59304 THEN PM_Q =
"second";
         IF PM25_AVG > 13.59304 AND PM25_AVG LE 14.75918 THEN PM_Q =
"third";
         IF PM25_AVG > 14.75918 THEN PM_Q = "highest";
         TEMP_C = TEMP_AVG - 272.15;
RUN;
PROC SORT DATA=PM_COG_CAT;
         BY PM_Q;
RUN;

***************************************
First, look at the descriptive statistics,
broken down by quantile of PM2.5 exposure.
```

```
***************************************;

* Continuous variables for the total sample;
PROC MEANS DATA=PM_COG_CAT;
	BY PM_Q;
	VAR AGE TEMP_C ASSESS_INTERVAL BMI;
RUN;

* Categorical variables;
PROC FREQ DATA=PM_COG_CAT;
	TABLES PM_Q*URBANGRP PM_Q*SEASON PM_Q*INCIDENT_IMPAIRMENT
PM_Q*STROKE1 PM_Q*RACE PM_Q*GENDER PM_Q*ED_CAT PM_Q*REGION
PM_Q*INCOME_4CAT PM_Q*SMOKE PM_Q*ALC_NIAAA PM_Q*EXERCISE_CAT
PM_Q*DEPRESSED PM_Q*DIAB_SRMED_GLU PM_Q*HYPER_SRMEDS_BP
PM_Q*LIPIDEMIA_MEDS_LABS;
RUN;

*********************************
Calculate the difference in impairment
across race
***********************************;
PROC FREQ DATA=PM_COG;
	TABLES INCIDENT_IMPAIRMENT*RACE;
RUN;

************************************
How many participants had estimated
exposure intervals less than 365 days?
************************************;
PROC MEANS DATA=PM_COG MEAN STD MEDIAN N min max;
	VAR DAY;
	WHERE DAY < 365;
RUN;

***************************************
What is different between participants
with less than 365 days of exposure data,
and those with at least 365 days of exposure
data?
**************************************;

* Create an indicator variable for having full exposure;
DATA PM_COG_EXP;
	SET PM_COG;
	IF DAY < 365 THEN FULL_EXPOSURE = "no"; ELSE FULL_EXPOSURE = "yes";
RUN;
```

```
* Perform t-tests on all continuous variables used in the fourth model for the main
analysis, as well as the day variable;
PROC TTEST DATA=PM_COG_EXP;
	CLASS FULL_EXPOSURE;
	VAR PM25_AVG TEMP_AVG_C ASSESS_INTERVAL DAY BMI;
RUN;
* Perform chi-square tests for all categorical variables used in the fourth model for
the main analysis.;
PROC FREQ DATA=PM_COG_EXP;
	TABLES FULL_EXPOSURE*SEASON FULL_EXPOSURE*STROKE1
FULL_EXPOSURE*GENDER FULL_EXPOSURE*RACE FULL_EXPOSURE*REGION
FULL_EXPOSURE*ED_CAT FULL_EXPOSURE*INCOME_4CAT
FULL_EXPOSURE*SMOKE FULL_EXPOSURE*ALC_NIAAA
FULL_EXPOSURE*EXERCISE_CAT FULL_EXPOSURE*DEPRESSED
FULL_EXPOSURE*DIAB_SRMED_GLU FULL_EXPOSURE*HYPER_SRMEDS_BP
FULL_EXPOSURE*LIPIDEMIA_MEDS_LABS/ CHISQ;
RUN;

***************************************
What's the distribution of race by
urbanicity?
***************************************;
PROC FREQ DATA=PM_COG;
	TABLES RACE*URBANGRP;
RUN;

***************************************
What's the distribution of stroke region
by urbanicity?
***************************************;
PROC FREQ DATA=PM_COG;
	TABLES REGION*URBANGRP;
RUN;
```
