# Supplementary material for: Fine Particulate Matter and Incident Cognitive Impairment in the REasons for Geographic and Racial Differences in Stroke (REGARDS) Cohort
Source: PLoS One. 2013 Sep 25;8(9):e75001. doi: 10.1371/journal.pone.0075001 (PMC3783452; doi:10.1371/journal.pone.0075001)
Supplement: Code S3 — Code for analyses. SAS program file detailing the analyses for the study, using logistic regression. (PDF) [file pone.0075001.s004.pdf]

```
*********************************************************
Author: Matthew Shane Loop

Purpose: This program will re-analyze the data
                for the pm and cognition data for the project. Details about
     the project can be found on the Regards and Environment
     Wiki page on the SSG Wiki.
********************************************************;
LIBNAME FINAL "..\data";
DATA PM_COG;
        SET FINAL.PM_COG;
RUN;

***************
MAIN ANALYSIS
***************;



*************************************************************************
This analysis will utilize logistic regression, with INCIDENT_IMPAIRMENT as
the outcome variable, to determine whether there was a relationship between
1-year average PM2.5 level prior to the baseline telephone interview and the
log odds of being impaired at the most recent follow-up. Specified a priori,
the following 4 models will be fit:
1. INCIDENT_IMPAIRMENT = PM2.5 + known confounders
2. INCIDENT_IMPAIRMENT = PM2.5 + known confounders + demographics
3. INCIDENT_IMPAIRMENT = PM2.5 + known confounders + demographics +
behavioral factors
4. INCIDENT_IMPAIRMENT = PM2.5 + known confounders + demographics +
behavioral factors + known comorbidites of cognitive impairment

Approximately 95% large-sample Wald confidence intervals will be constructed for
at
10 microgram/m^3 change in PM2.5 levels.
*************************************************************************;

* Model 1;
PROC LOGISTIC DATA=PM_COG DESC;
        CLASS SEASON;
        MODEL INCIDENT_IMPAIRMENT = PM25_AVG TEMP_AVG_C SEASON
STROKE1 ASSESS_INTERVAL / CLODDS=BOTH;
        UNITS PM25_AVG = 10;
        OUTPUT OUT=MODEL1 PREDICTED=PHAT;
RUN;QUIT;
```

```
* Model 2;
PROC LOGISTIC DATA=PM_COG DESC;
        CLASS SEASON GENDER RACE REGION ED_CAT INCOME_4CAT;
        MODEL INCIDENT_IMPAIRMENT = PM25_AVG TEMP_AVG_C SEASON
STROKE1 ASSESS_INTERVAL GENDER RACE REGION ED_CAT INCOME_4CAT/
CLODDS=BOTH;
        UNITS PM25_AVG = 10;
RUN;QUIT;

* Model 3;
PROC LOGISTIC DATA=PM_COG DESC;
        CLASS SEASON GENDER RACE REGION ED_CAT INCOME_4CAT SMOKE
EXERCISE_CAT ALC_NIAAA;
        MODEL INCIDENT_IMPAIRMENT = PM25_AVG TEMP_AVG_C SEASON
STROKE1 ASSESS_INTERVAL GENDER RACE REGION ED_CAT INCOME_4CAT
SMOKE ALC_NIAAA EXERCISE_CAT BMI/ CLODDS=BOTH;
        UNITS PM25_AVG = 10;
RUN;QUIT;

* Model 4;
PROC LOGISTIC DATA=PM_COG DESC;
        CLASS SEASON GENDER RACE REGION ED_CAT INCOME_4CAT SMOKE
EXERCISE_CAT ALC_NIAAA DIAB_SRMED_GLU HYPER_SRMEDS_BP
LIPIDEMIA_MEDS_LABS;
        MODEL INCIDENT_IMPAIRMENT = PM25_AVG TEMP_AVG_C SEASON
STROKE1 ASSESS_INTERVAL GENDER RACE REGION ED_CAT INCOME_4CAT
SMOKE ALC_NIAAA EXERCISE_CAT BMI DEPRESSED DIAB_SRMED_GLU
HYPER_SRMEDS_BP LIPIDEMIA_MEDS_LABS/ CLODDS=BOTH;
        UNITS PM25_AVG = 10;
RUN;QUIT;

*****************
Interaction between urbanicty and PM2.5 exposure.
*****************;

* Interaction between urbanicity and PM2.5 in model 1;
PROC LOGISTIC DATA=PM_COG DESC;
        CLASS SEASON URBANGRP;
        MODEL INCIDENT_IMPAIRMENT = PM25_AVG URBANGRP
PM25_AVG*URBANGRP TEMP_AVG_C SEASON STROKE1 ASSESS_INTERVAL /
CLODDS=BOTH;
        UNITS PM25_AVG = 10;
        ODDSRATIO PM25_AVG;
        OUTPUT OUT=URBAN PREDICTED=PHAT;
RUN;QUIT;
```

```
* Interaction between urbanicity and PM2.5 in model 2;
PROC LOGISTIC DATA=PM_COG DESC;
	CLASS SEASON URBANGRP GENDER RACE REGION ED_CAT INCOME_4CAT;
	MODEL INCIDENT_IMPAIRMENT = PM25_AVG URBANGRP
PM25_AVG*URBANGRP TEMP_AVG_C SEASON STROKE1 ASSESS_INTERVAL
GENDER RACE REGION ED_CAT INCOME_4CAT/ CLODDS=BOTH;
	UNITS PM25_AVG = 10;
	ODDSRATIO PM25_AVG;
	OUTPUT OUT=URBAN PREDICTED=PHAT;
RUN;QUIT;

* Interaction between urbanicity and PM2.5 in model 3;
PROC LOGISTIC DATA=PM_COG DESC;
	CLASS SEASON URBANGRP GENDER RACE REGION ED_CAT INCOME_4CAT
SMOKE ALC_NIAAA EXERCISE_CAT;
	MODEL INCIDENT_IMPAIRMENT = PM25_AVG URBANGRP
PM25_AVG*URBANGRP TEMP_AVG_C SEASON STROKE1 ASSESS_INTERVAL
GENDER RACE REGION ED_CAT INCOME_4CAT SMOKE ALC_NIAAA EXERCISE_CAT
BMI/ CLODDS=BOTH;
	UNITS PM25_AVG = 10;
	ODDSRATIO PM25_AVG;
	OUTPUT OUT=URBAN PREDICTED=PHAT;
RUN;QUIT;

* Interaction between urbanicity and PM2.5 in model 4;
PROC LOGISTIC DATA=PM_COG DESC;
	CLASS SEASON URBANGRP GENDER RACE REGION ED_CAT INCOME_4CAT
SMOKE ALC_NIAAA EXERCISE_CAT DIAB_SRMED_GLU HYPER_SRMEDS_BP
LIPIDEMIA_MEDS_LABS;
	MODEL INCIDENT_IMPAIRMENT = PM25_AVG URBANGRP
PM25_AVG*URBANGRP TEMP_AVG_C SEASON STROKE1 ASSESS_INTERVAL
GENDER RACE REGION ED_CAT INCOME_4CAT SMOKE ALC_NIAAA EXERCISE_CAT
BMI DEPRESSED DIAB_SRMED_GLU HYPER_SRMEDS_BP LIPIDEMIA_MEDS_LABS/
CLODDS=BOTH;
	UNITS PM25_AVG = 10;
	ODDSRATIO PM25_AVG;
	OUTPUT OUT=URBAN PREDICTED=PHAT;
RUN;QUIT;

*********************
POST-HOC ANALYSIS
*********************;

*************************************************************************************
********
```

Sensitivity analysis: Use incident_impairment2 as outcome. This variable indicates whether
or not the participant was impaired on both of the most recent two assessments. Naturally,
we had to include participants who had at least 3 total assessments (1 baseline, then two
follow-up assessments).
```
*****************************************************************************************;
* Model 1;
PROC LOGISTIC DATA=PM_COG DESC;
	CLASS SEASON;
	MODEL INCIDENT_IMPAIRMENT2 = PM25_AVG TEMP_AVG_C SEASON
STROKE1 ASSESS_INTERVAL / CLODDS=BOTH;
	WHERE TOTAL_ASSESS > 2;
	UNITS PM25_AVG = 10;
RUN;QUIT;

* Model 2;
PROC LOGISTIC DATA=PM_COG DESC;
	CLASS SEASON GENDER RACE REGION ED_CAT INCOME_4CAT;
	MODEL INCIDENT_IMPAIRMENT2 = PM25_AVG TEMP_AVG_C SEASON
STROKE1 ASSESS_INTERVAL GENDER RACE REGION ED_CAT INCOME_4CAT/
CLODDS=BOTH;
	WHERE TOTAL_ASSESS > 2;
	UNITS PM25_AVG = 10;
RUN;QUIT;

* Model 3;
PROC LOGISTIC DATA=PM_COG DESC;
	CLASS SEASON GENDER RACE REGION ED_CAT INCOME_4CAT SMOKE
EXERCISE_CAT ALC_NIAAA;
	MODEL INCIDENT_IMPAIRMENT2 = PM25_AVG TEMP_AVG_C SEASON
STROKE1 ASSESS_INTERVAL GENDER RACE REGION ED_CAT INCOME_4CAT
SMOKE ALC_NIAAA EXERCISE_CAT BMI/ CLODDS=BOTH;
	WHERE TOTAL_ASSESS > 2;
	UNITS PM25_AVG = 10;
RUN;QUIT;

* Model 4;
PROC LOGISTIC DATA=PM_COG DESC;
	CLASS SEASON GENDER RACE REGION ED_CAT INCOME_4CAT SMOKE
EXERCISE_CAT ALC_NIAAA DIAB_SRMED_GLU HYPER_SRMEDS_BP
LIPIDEMIA_MEDS_LABS;
	MODEL INCIDENT_IMPAIRMENT2 = PM25_AVG TEMP_AVG_C SEASON
STROKE1 ASSESS_INTERVAL GENDER RACE REGION ED_CAT INCOME_4CAT
```

```
SMOKE ALC_NIAAA EXERCISE_CAT BMI DEPRESSED DIAB_SRMED_GLU
HYPER_SRMEDS_BP LIPIDEMIA_MEDS_LABS/ CLODDS=BOTH;
        WHERE TOTAL_ASSESS > 2;
        UNITS PM25_AVG = 10;
RUN;QUIT;

*********************************************************************************
********
Determine whether the removal of the participants who had less than 365 days of exposure
data affects the main analysis.
*********************************************************************************
********;

* Model 1;
PROC LOGISTIC DATA=PM_COG DESC;
        CLASS SEASON;
        MODEL INCIDENT_IMPAIRMENT = PM25_AVG TEMP_AVG_C SEASON
STROKE1 ASSESS_INTERVAL / CLODDS=BOTH;
        WHERE DAY GE 365;
        UNITS PM25_AVG = 10;
RUN;QUIT;

* Model 2;
PROC LOGISTIC DATA=PM_COG DESC;
        CLASS SEASON GENDER RACE REGION ED_CAT INCOME_4CAT;
        MODEL INCIDENT_IMPAIRMENT = PM25_AVG TEMP_AVG_C SEASON
STROKE1 ASSESS_INTERVAL GENDER RACE REGION ED_CAT INCOME_4CAT/
CLODDS=BOTH;
        WHERE DAY GE 365;
        UNITS PM25_AVG = 10;
RUN;QUIT;

* Model 3;
PROC LOGISTIC DATA=PM_COG DESC;
        CLASS SEASON GENDER RACE REGION ED_CAT INCOME_4CAT SMOKE
EXERCISE_CAT ALC_NIAAA;
        MODEL INCIDENT_IMPAIRMENT = PM25_AVG TEMP_AVG_C SEASON
STROKE1 ASSESS_INTERVAL GENDER RACE REGION ED_CAT INCOME_4CAT
SMOKE ALC_NIAAA EXERCISE_CAT BMI/ CLODDS=BOTH;
        WHERE DAY GE 365;
        UNITS PM25_AVG = 10;
RUN;QUIT;

* Model 4;
PROC LOGISTIC DATA=PM_COG DESC;
```

```
	CLASS SEASON GENDER RACE REGION ED_CAT INCOME_4CAT SMOKE
EXERCISE_CAT ALC_NIAAA DIAB_SRMED_GLU HYPER_SRMEDS_BP
LIPIDEMIA_MEDS_LABS;
	MODEL INCIDENT_IMPAIRMENT = PM25_AVG TEMP_AVG_C SEASON
STROKE1 ASSESS_INTERVAL GENDER RACE REGION ED_CAT INCOME_4CAT
SMOKE ALC_NIAAA EXERCISE_CAT BMI DEPRESSED DIAB_SRMED_GLU
HYPER_SRMEDS_BP LIPIDEMIA_MEDS_LABS/ CLODDS=BOTH;
	WHERE DAY GE 365;
	UNITS PM25_AVG = 10;
RUN;QUIT;
```
